# How much is tuberculosis screening worth? Estimating the value of active case finding for tuberculosis in South Africa, China, and India

**DOI:** 10.1186/s12916-014-0216-0

**Published:** 2014-10-30

**Authors:** Andrew S Azman, Jonathan E Golub, David W Dowdy

**Affiliations:** Department of Epidemiology, Johns Hopkins Bloomberg School of Public Health, 615 North Wolfe Street, Baltimore, MD 21205 USA; Center for Tuberculosis Research, Department of Medicine, Division of Infectious Diseases, Johns Hopkins School of Medicine, 1550 Orleans St., Baltimore, MD 21231 USA

**Keywords:** Mathematical Modeling, Screening, Cost-Effectiveness, Active Case Finding, Tuberculosis, TB

## Abstract

**Background:**

Current approaches are unlikely to achieve the aggressive global tuberculosis (TB) control targets set for 2035 and beyond. Active case finding (ACF) may be an important tool for augmenting existing strategies, but the cost-effectiveness of ACF remains uncertain. Program evaluators can often measure the cost of ACF per TB case detected, but how this accessible measure translates into traditional metrics of cost-effectiveness, such as the cost per disability-adjusted life year (DALY), remains unclear.

**Methods:**

We constructed dynamic models of TB in India, China, and South Africa to explore the medium-term impact and cost-effectiveness of generic ACF activities, conceptualized separately as discrete (2-year) campaigns and as continuous activities integrated into ongoing TB control programs. Our primary outcome was the cost per DALY, measured in relationship to the cost per TB case actively detected and started on treatment.

**Results:**

Discrete campaigns costing up to $1,200 (95% uncertainty range [UR] 850–2,043) per case actively detected and started on treatment in India, $3,800 (95% UR 2,706–6,392) in China, and $9,400 (95% UR 6,957–13,221) in South Africa were all highly cost-effective (cost per DALY averted less than per capita gross domestic product). Prolonged integration was even more effective and cost-effective. Short-term assessments of ACF dramatically underestimated potential longer term gains; for example, an assessment of an ACF program at 2 years might find a non-significant 11% reduction in prevalence, but a 10-year evaluation of that same intervention would show a 33% reduction.

**Conclusions:**

ACF can be a powerful and highly cost-effective tool in the fight against TB. Given that short-term assessments may dramatically underestimate medium-term effectiveness, current willingness to pay may be too low. ACF should receive strong consideration as a basic tool for TB control in most high-burden settings, even when it may cost over $1,000 to detect and initiate treatment for each extra case of active TB.

**Electronic supplementary material:**

The online version of this article (doi:10.1186/s12916-014-0216-0) contains supplementary material, which is available to authorized users.

## Background

Global targets for tuberculosis (TB) control now include a 95% reduction in TB deaths and less than 10 cases per 100,000 population by 2035 [1]. Such targets will not be met without strategies to diagnose and treat people with active TB earlier in their disease course [2-4]. Recent World Health Organization (WHO) guidelines recommend, for the first time, routine TB screening of certain high-risk groups (e.g., people living with HIV) [5], and active TB case finding is increasingly becoming part of an essential package of TB prevention and care. However, with limited resources available to improve health worldwide, it is critical to implement those interventions likely to provide greatest impact and value for money.

Although passive (symptom-driven) diagnosis and treatment of sputum smear-positive TB is among the most cost-effective health interventions available, most economic evaluations of TB interventions have not, to date, included active TB case finding [6]. As such, the potential impact and cost-effectiveness of active case finding (ACF) remains largely unknown. Recently, a large community-randomized trial in Zambia and South Africa found non-significant reductions in community-wide TB prevalence and incidence from a household-based contact investigation intervention, and no impact of community-based enhanced case-finding, but was only powered to detect a very large effect (30% prevalence reduction over 4 years) [7]. Similarly, a systematic review of earlier evidence concluded that the population-level effect of active TB case finding remains uncertain [8].

**Figure 1:**
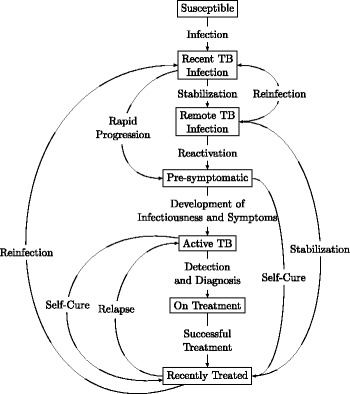
**Schematic of compartmental TB transmission model.** Boxes represent states in the model (HIV not shown but a more complete diagram can be found in Additional file 1: Figure S1) and arrows represent flows between the states. We model active TB case finding as a one-time increase in the rate of “Detection and Diagnosis,” which incorporates all efforts from screening to initiation of therapy but does not detect pre-symptomatic cases.

Though population-level reduction in TB incidence and prevalence has been difficult to demonstrate empirically, active case-finding initiatives could nevertheless reduce TB transmission to an important degree. If such reductions in transmission can be achieved, they could also generate cost savings for TB control programs, making ACF potentially both epidemiologically relevant and cost-effective in the medium-term (10 years), even if shorter-term research studies cannot detect a population-level effect. In this setting of empirical uncertainty, mathematical models can provide “best available evidence” estimates [9]. Here, we use combined transmission-economic models of TB epidemics in China, India, and South Africa (Figure 1) to estimate the most likely medium-term epidemiologic impact and cost-effectiveness of feasible case-finding approaches. By modeling generic interventions, we create a tool for converting data that are easily estimable by people considering specific case-finding programs (i.e., program costs and number of additional TB cases detected from ACF campaigns using a specific approach) into data that are important for decision making (i.e., cost per disability adjusted life year (DALY) averted). We use these results to provide guidance as to how much donors and in-country TB control programs should be willing to pay to find one additional case of active TB.

## Methods

### TB transmission model

We developed a compartmental model of adult TB transmission represented by a system of ordinary differential equations (Figure 1, Table 1, Additional file 1). This model follows the basic structure of other widely-used TB transmission models, but incorporates additional structure to account for early disease stages that do not involve sufficient symptomatic severity (e.g., a prolonged cough) to drive patients to seek care [10]. We assume that individuals in these early disease stages – as well as anyone whose disease never progresses to the point of detectability by sputum smear – are less infectious than individuals with smear-positive pulmonary TB [2-4,11]. We divide latent TB into two non-infectious classes to reflect the increased risk of TB progression soon after (re-)infection [5,12]. We assume that, for patients who are diagnosed and started on therapy, treatment immediately renders TB as non-infectious and lasts an average of 6 months, during which reinfection does not occur. We also incorporate an increased risk of relapse for an average of 2 years after treatment [6,13]. For simplicity, we do not explicitly consider population growth or migration. Source code for the model and analyses from this manuscript, written in R version 3.0.1 (R Foundation for Statistical Computing), is available from github.com [14].

**Table 1:** **Key transmission model parameters**

**Parameter description**	**HIV** ^**−**^	**HIV** ^**+**^_**CD4 ≥350**_	**HIV** ^**+**^_**CD4 <350**_ **no ART**	**HIV** ^**+**^ **on ART**	**Source**
Number of TB infections per smear-positive case^§^ (yr^−1^)	16.16/11.16/23.63	16.16/11.16/23.63	16.16/11.16/23.63	16.16/11.16/23.63	Fit
Relative transmissibility of smear-negative TB	0.22	0.22	0.22	0.22	[11,15]
Relative risk of reinfection when latent infected	0.21	0.45	1.00	0.45	[7,13,16]
Relative transmissibility of pre-symptomatic TB	0.22	0.22	0.22	0.22	Assumed
Rate of stabilization^¶^ (yr^−1^)	0.50	0.50	0.50	0.50	[12,17]
Rate of stabilization after successful treatment (yr^−1^)	0.20	0.20	0.20	0.20	[12]
Duration of treatment (yr)	0.50	0.50	0.50	0.50	[8,18]
Rate of rapid progression to active TB after recent infection (yr^−1^)	0.07	0.26	0.70	0.26	[12,19]
Rate of endogenous reactivation to active TB after remote infection (yr^−1^)	0.49 × 10^-4^	0.024	0.08	0.024	[20]
Relapse rate (yr^−1^)	0.02	0.02	0.02	0.02	Assumed
Duration of pre-symptomatic TB (yr)	0.75	0.25	0.10	0.25	Assumed
Proportion of pulmonary TB that is smear-positive	0.75	0.65	0.40	0.65	[18]
Proportion of TB that is extra-pulmonary	0.15	0.23	0.40	0.23	Assumed
Mortality rate from smear-positive TB (yr^−1^)	0.23	0.56	1.33	0.56	[21]
Mortality rate from smear-negative and extra pulmonary TB (yr^−1^)	0.18	0.53	1.33	0.53	[21]
Self-cure rate, smear-positive (yr^−1^)	0.10	0.07	0.00	0.07	[21]
Self-cure rate, smear-negative and extra-pulmonary (yr^−1^)	0.15	0.11	0.00	0.11	[21]
Detection (and diagnosis) rate (yr^−1^) ^§^	1.01/1.05/1.94	1.01/1.05/1.94	1.01/1.05/1.94	1.01/1.05/1.94	fit
Rate of new HIV infections (yr^−1^)^§^	0.34e-3/0.39e-6/0.02	0.00	0.00	0.00	fit
Mean time from HIV infection to CD4 count of 350 (yr)	0.00	4.19	0.00	0.00	[22]
Rate of progression from ART eligibility to on ART (yr^−1^)^§^	0.00	0.00	0.04/0.06/0.19	0.00	fit
Mortality rate from HIV (yr^−1^)	0.00	0.01	0.13	0.04	[23]

^§^Fitted values shown for India/China/South Africa.

^¶^Stabilization refers to the rate of transition between a fast latent phase (“recent infection”) and a slow latent phase (“remote infection”).

Columns represent the parameter values for different HIV classes with the final column indicating the source for the parameter assumption.

ART, Antiretroviral therapy; TB, Tuberculosis.

As the focus of our inference is on TB, we choose a simple representation of HIV transmission with compartments for individuals who are HIV-uninfected, HIV-infected with CD4 count ≥350 cells/mm^3^, HIV-infected with CD4 count <350 cells/mm^3^ and not on antiretroviral therapy (ART), and HIV-infected on ART. Rather than explicitly modeling HIV transmission, we assume that new HIV infections occur at a constant rate, as does progression of CD4 decline and, among those with CD4 < 350 cells/mm^3^, ART initiation. We assume that individuals who are either on ART or have CD4 count ≥350 cells/mm^3^ can be characterized using a weighted average of the attributes associated with no HIV infection (70%) and CD4 count <350 not on ART (30%) (Additional file 1). Our approach to parameterizing HIV states within this model is similar to previously published data-driven and modeling analyses [23,24].

### Model calibration

We calibrated models separately to published data in each country (Table 2) by iteratively fitting the TB and HIV components of the model. In the TB components, we fit the rates of TB transmission and detection to estimates of TB incidence and case detection, respectively. In the HIV components, we fit the rates of HIV infection and ART initiation to estimates of adult HIV prevalence and population ART coverage. To capture the decreasing trend in TB incidence in India and China, we first fit the model to steady state in 2004 based on data from the WHO [7,18], then solved for a constant rate of decrease in the transmission rate yielding a population with the target 2011 incidence (Table 2).

**Table 2:** **Key epidemiologic and economic variables for representative communities in China, India, and South Africa**

	**China** ^**‡**^	**India** ^**‡**^	**South Africa**
TB incidence 2011 (per 100,000) [18]	75	181	993
TB case detection proportion [18]	0.67	0.65^§^	0.69
Adult HIV prevalence (per 100) [25]	0.058	0.4	17.3
Adult ART coverage (%) [25,26]	50	40	75
Per capita GDP (2011 USD)	5,439	1,528	8,090
Cost of first-line treatment [18]	1,029	81	232

^**‡**^These countries were fit to data from 2004 then fit to 2011 incidence by adjusting the transmission parameter.

^§^Used the upper bound of the 95% confidence interval of the 2011 case detection proportion from [18] to account for cases seen and treated in the private sector.

ART, Antiretroviral therapy; TB, Tuberculosis.

### Intervention

The goal of this analysis was to evaluate the appropriate cost per additional case detected through ACF, not to evaluate the precise impact and cost-effectiveness of any specific intervention activity. We assume that the cost of an ACF campaign, as well as the additional number of TB cases diagnosed and treated, can be locally measured (or estimated) for any given campaign. Thus, we consider ACF in its most straightforward representation through a one-time increase in the rate of transition from active TB to “on treatment” (Figure 1). We refer to this rate as the detection rate, though it incorporates detection, diagnosis, and initiation of appropriate therapy. We separately simulated discrete ACF campaigns lasting 2 years (ending immediately thereafter), and programmatic changes incorporating ACF into routine TB control activities for the duration of the analysis period (10 years). We conservatively assume that those in the pre-symptomatic stage cannot be detected by ACF. For the main analyses, roughly following the proportion of smear-positive TB cases detected through enhanced case finding in the ZAMSTAR study (29.7%) [7], we consider interventions that would increase the number of cases detected during the first intervention year by 25% of the 2011 counterfactual/baseline. After the first year, the number of additional cases detected falls, as a constant detection rate is applied to a smaller prevalent TB pool.

### Economic evaluation

We calculate TB-specific DALYs as the sum of years of life lost plus years of life with disability over a time horizon of one to ten years. To estimate years of life with disability, we use disability weights for TB and HIV from the Global Burden of Disease Study 2010 [27]. To relate the cost per case detected by ACF to its cost-effectiveness, we calculate the number of cases detected and treated under the counterfactual (no intervention) scenario and compare this to the number of cases detected and treated with ACF in place. We then calculate the incremental cost of the intervention as:

$$ incremental\kern0.5em  intervention\kern0.5em  cost=\left( incremental\kern0.5em  number\kern0.5em  of case s\kern0.5em  detected\right)\times \left( cost\kern0.5em  per\kern0.5em  case\kern0.5em  detected+ cost\kern0.5em  per\kern0.5em  case\kern0.5em  treated\right), $$

and the incremental cost-effectiveness ratio (ICER, expressed in year 2012 US dollars per DALY averted) as:

$$ ICER=\left( incremental\kern0.5em  interventions\kern0.5em  \cos t\right)/\left( incremental\kern0.5em  number\kern0.5em  of\kern0.5em  DALYs\kern0.5em  averted\right). $$

Thus, after estimating the number of incremental cases detected and the incremental DALYs averted from the transmission model, and taking estimates of the cost per case treated, we can calculate the ICER as a function of the cost per case detected (Additional file 1).

We adopt the perspective of the national TB program (assumed to be responsible for ACF campaigns and treatment of TB, but not HIV) and discount all future costs and health outcomes at 3% per year [28,29]. Since the cost-effectiveness of ACF depends strongly on the analytic time horizon (becoming less cost-effective if future cases averted are ignored), we vary the time horizon from 1 to 10 years. Thus, for 2-year campaigns, we consider effects for up to 8 years after the campaign ends. We define interventions with an incremental cost per DALY averted less than 2012 per capita gross domestic product (GDP) as highly cost-effective [29].

### Sensitivity and uncertainty analysis

We conducted one-way sensitivity analyses for key model parameters with a focus on the cost-effectiveness threshold (i.e., maximum cost per-case detected for an ICER equal to each country’s per capita GDP) for 2-year campaigns at 2-, 5-, and 10-year analytic horizons. We also conducted multivariate uncertainty analyses by selecting 20,000 parameter sets from independent prior beta distributions using Latin Hypercube sampling (Additional file 1) [30]. We calculated the cost-effectiveness thresholds from each run and used the 2.5^th^ and 97.5^th^ percentiles as the 95% uncertainty ranges. To compare the effect of each parameter, adjusted for all other parameters, we calculated partial rank correlation coefficients from the multivariate uncertainty analyses [30].

## Results

We simulated ACF programs in representative communities of China, India, and South Africa. ACF interventions that increased the number of cases diagnosed and treated by 25% in their first year (i.e., an additional 13 cases detected in a community of 100,000 in China, 31 per 100,000 in India, and 171 per 100,000 in South Africa) reduced the average duration of untreated disease from 15.2 to 12.7 months in South Africa, 20.0 to 17.3 months in India, and 20.4 to 17.0 months in China.

### Discrete ACF campaigns

**Figure 2:**
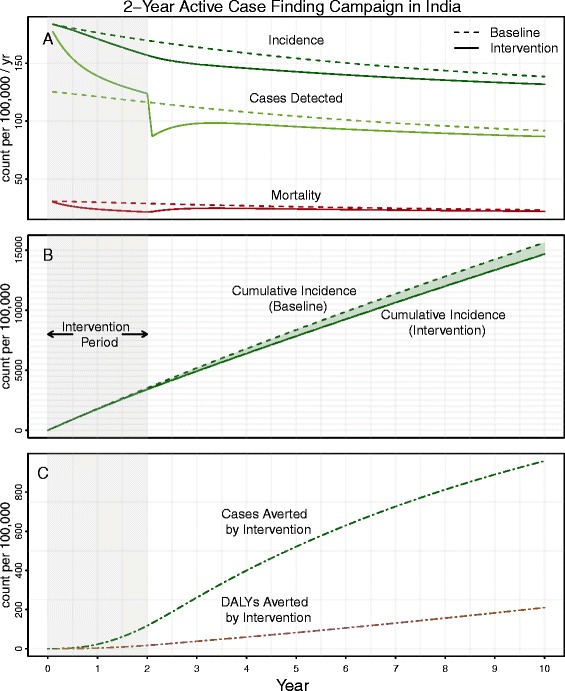
**Impact of a discrete 2-year active case finding campaign in India.** Panel **A** illustrates the incidence rate (dark green), case detection rate (light green), and mortality rate (red) for a baseline/counterfactual scenario (dashed) compared against an intervention scenario (solid) in which TB case detection is increased, through active case finding, by 25% from the cases detected in the first year (2012). Panel **B** shows the cumulative incidence (per 100,000) for both the intervention (solid) and baseline (dashed) scenarios with the area between the two curves representing the cases averted through active case finding. Panel **C** shows the cases averted by the intervention (green), and DALYs averted by the intervention (brown) – a function of cases averted and mortality averted by intervention. The grey shading highlights the component of the intervention effect that would be observable during the course of a 2-year intervention study.

Two-year ACF campaigns of this magnitude had small but important population-level effects. In representative communities of 1 million individuals in India, China, and South Africa, respectively, a campaign that increased case finding by 25% in year one and ended after 2 years could avert 277 (95% uncertainty range [UR] 173–557), 100 (95% UR 63–201), and 2,165 (95% UR 1,504-3,307) deaths over 10 years, with 39 to 56% of deaths occurring during the time period of the intervention (Figure 2). Even in South Africa, however, this would correspond to only a 1.4% reduction in all-cause mortality during the intervention (1,548 averted deaths of a total 1,123,567 expected deaths [31]); an unfeasibly large study would be required to detect the effect of this rare disease. In contrast to effects on mortality, effects of ACF on incidence and DALYs were largely felt not during the study period but after the end of the 2-year campaign, reflecting known delays between transmission and detectable disease. Of the cumulative cases averted by ACF over 10 years in an Indian community, less than one in eight (12%) occurred during the intervention timeframe (Figure 2B,C). Thus, an evaluation conducted over the course of the 2-year intervention, not considering future effects, would underestimate medium-term (10-year) impact on incidence by over 85%. Results were similar in communities in China and South Africa (Additional file 1).

**Figure 3:**
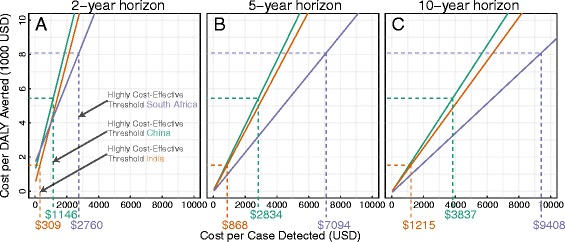
**Thresholds for discrete active TB case finding campaigns to be highly cost-effective in South Africa, China, and India, by cost per case detected and analytic time horizon.** Each solid line shows the incremental cost per DALY averted (y-axis, 1,000 USD units), as a function of the cost per case detected and started on treatment (x-axis). The dashed lines and corresponding numbers below the x-axis show the cost per case detected that corresponds to the “highly cost-effective” threshold in India (orange), China (green), and South Africa (purple). Panels **A–C** show these relationships for the same intervention, but under different time horizons; Panel **A** considers only effects that occur in the first 2 years (i.e., ignoring longer-term effects), whereas panels **B** and **C** consider costs and effects over 5 and 10 years, respectively.

From an economic perspective, ACF campaigns – even those lasting only 2 years – were highly cost-effective across a range of scenarios. Specifically, 2-year ACF campaigns that cost $1,200 (95% UR 850–2,043) per case detected (and started on treatment) in India, $3,800 (95% UR 2,706–6,392) per case detected in China, and $9,400 (95% UR 6,957–13,221) per case detected in South Africa were all “highly cost-effective” by traditional standards (cost per DALY averted less than per capita GDP) over a 10-year time horizon (Figure 3C, Additional file 1) [29]. However, failure to account for future effects reduced these cost-effectiveness thresholds by nearly 75% (Figure 3A). For example, to be considered highly cost-effective in India using a 2-year time horizon, an ACF campaign would need to cost under $300 (95% UR 275–343) per case detected.

### Sustained ACF programs

**Figure 4:**
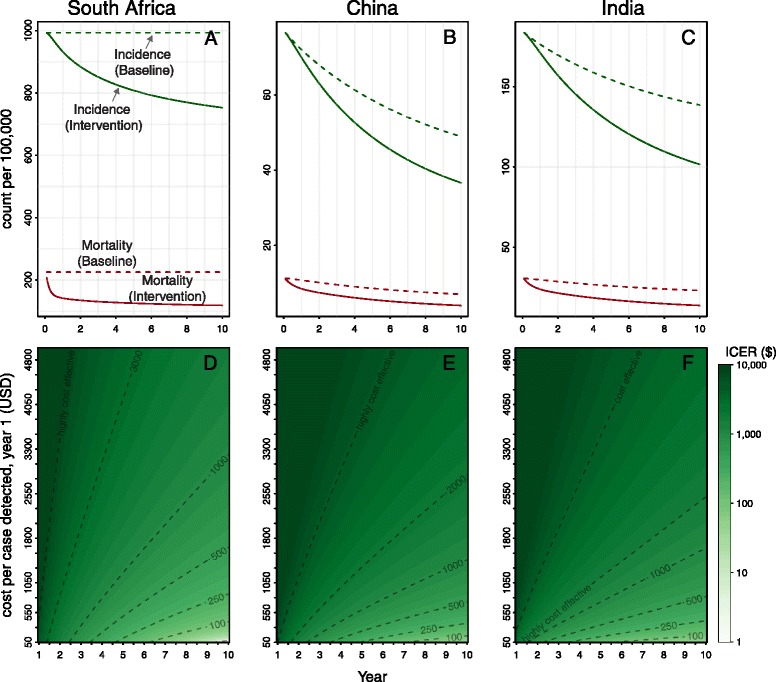
**Epidemiologic and economic impact of sustained active case finding over 10 years.** Panels **A–C** (top row) shows projected incidence (green) and mortality (red) in communities with a sustained active case finding intervention capable of increasing the cases detected in the first year by 25% of the counterfactual scenario (solid line) and a counterfactual scenario with no intervention (dashed line). Panels **D–F** (second row) shows the corresponding cost-effectiveness of the intervention as a function of the cost per case detected in year 1 (y-axis) and the time horizon over which costs and effects are considered (x-axis, Note: the time horizon here is equal to the duration of the intervention in this sustained setting). Contour lines are labeled in these plots as the cost per DALY averted, with “highly cost-effective” corresponding to a country’s per-capita GDP.

Unlike short-term campaigns, sustained ACF programs over 10 years showed dramatic population-level impact on both incidence (22 to 27% reduction) and mortality (40 to 44% reduction) (Figure 4A). Owing to compounded impact on transmission over time, such sustained campaigns are also substantially more cost-effective; by seven years, sustained campaigns costing even as much as $5,000 per case detected were projected to be highly cost-effective in two of three scenarios (Figure 4D–F, top of y-axis). A sustained ACF intervention in India would reduce TB prevalence by only 11% after 2 years, but could reduce prevalence by 33% within 10 years (Additional file 1). Similarly, only about one of eight deaths averted would occur during the first 2 years.

### Sensitivity analyses

The estimated cost per DALY averted in each setting was robust to the intensity of the ACF intervention. Varying the increase in number of cases detected in year one from 5% to 50% had little effect (<3.5% change, Additional file 1: Section 4 and Figure S2) on the estimated cost per DALY averted, as long as the cost per additional case detected and treated remained constant. Thus, while larger ACF campaigns had greater impact (and greater cost), the relationship between cost and impact was not strongly dependent on campaign size. The cost-effectiveness thresholds in all three communities were robust to parameter selection in both one-way and multivariable uncertainty analyses. The rate of rapid progression to active TB after recent infection, the transmission rate, and the detection rate (all in HIV-uninfected classes) had the largest effect on ACF cost-effectiveness in India (Additional file 1). In South Africa, where HIV plays a critical role in TB epidemiology, the transmission rate, the smear-negative TB mortality rate amongst HIV-infected individuals with CD4 < 350 not on ART, and the detection rate, had the largest effects (Additional file 1).

## Discussion

Although the impact of ACF campaigns on population-level epidemiology remains empirically uncertain, this model demonstrates that campaigns of feasible intensity can be highly cost-effective and exert important population-level effects – effects that even large studies may be incapable of detecting. For example, a 2-year case-finding campaign in India that increased case detection by 25% at a cost of $500 per case (detected and started on treatment) might, over 10 years, avert 960 TB cases, 2,100 DALYs, and save 280 lives in a city of one million people – at a cost of $620 per DALY averted, far less than India’s per-capita GDP. However, a study that assessed outcomes after 2 years would detect less than 15% of the epidemiological impact and would overestimate the long-term cost per DALY averted by a factor of four. In summary, rapid action is needed if TB control targets for 2035 are to be reached [32], and ACF may have strong population-level benefits in this time frame, but even large short-term studies are unlikely to detect those effects. By demonstrating these realities *in silico*, we argue for higher prioritization of active TB case finding on the global health agenda.

To date, empirical evidence of the population-level effect of ACF has been sparse and conflicting [8]. As a result, enthusiasm for ACF as a tool for population-level TB control is muted. Our results demonstrate that short-term evaluations are unlikely to correlate with long-term gains. For example, our model of sustained ACF in a representative Indian community was projected to reduce TB prevalence by 11% after 2 years – far lower than that seen in Zimbabwe using a mobile-van approach [15], and well within the confidence intervals of both arms of the ZAMSTAR trial, which has been cited as evidence of no population-level benefit of ACF [7]. However, after 10 years, this same intervention could reduce TB prevalence by a projected 33%; shorter-term studies would not detect this important population-level effect without a prolonged follow-up period.

Our results, consistent with a previous theoretical model [33], also suggest that our current willingness to pay for active TB case finding may be too low. For example, the Stop TB Partnership initially set a limit of $350 per smear-positive case found and started on treatment as a benchmark for grants through the TB REACH mechanism. Our analysis suggests that, in countries like China and South Africa, national TB programs should be willing to pay (at the WHO “highly cost-effective” threshold) 10 to 40 times as much per case detected and treated. A recent summary of 28 different ACF programs across 12 high-burden countries estimated that 17,236 additional smear-positive cases (relative to control populations) were detected at a cost of $14.9 million, or an average cost per smear positive case detected of $865 [34]. If these cases were linked to treatment, our model suggests that the average ACF campaign would be highly cost-effective in a setting like India within a 5-year horizon, and in settings like China or South Africa within a 2-year horizon. To the extent that additional smear-negative cases were also diagnosed and treated without additional expense, ACF would be even more cost-effective. Active TB case finding may compare favorably in cost-effectiveness terms to other widely implemented health interventions. For example, other models project that ART for HIV may cost $500 to $2,000 per DALY averted in most settings [17]. Our model suggests that a “best available evidence” approach might place TB screening programs costing $1,000 per case detected in the same basket of essential services as ART.

As with any model-based analysis, our findings are subject to certain limitations. We sought to simulate a generic ACF intervention in multiple countries without specifying the details of the target population or the case finding strategy. While we based the main intervention on an estimate from a large trial in South Africa [7], this is likely an upper limit on its impact. In sensitivity analyses (Additional file 1: Section 4 and Figure S2) we show that cost per DALY averted is relatively constant for different ACF campaign sizes, as long as the cost per additional case detected and treated remains constant. Campaigns that detected more cases had greater impact, but (for a given cost per additional case detected and treated) also had greater cost, and the relationship between cost per additional case detected/treated and cost per DALY averted was robust to the campaign size (Additional file 1: Figure S2). Thus, we expect that our findings would hold even if our estimate of ZAMSTAR’s impact was overly optimistic. If interventions are targeted to key populations (e.g., household contacts) or areas of high local transmission, even more favorable cost-effectiveness ratios may be achievable. Individuals detected through ACF approaches may have mild or asymptomatic illness and thus be less likely to complete their full course of medication. Though a recent review suggests that treatment outcomes are similar [8], others have suggested worse adherence among such cases [19]. We present our results in terms of cost per case detected in the first year of the campaign in order to allow for comparisons between countries and to be consistent with metrics used by the donor community. This specification, however, requires knowledge of the costs (fixed and variable) and case yields of a campaign *a priori*. Finally, we adopted a simple modeling approach in order to maximize transparency and generalizability, including fitting our models to communities that are representative of WHO notification data. More detailed data, and data from other settings, could be integrated to provide more locale-specific estimates in the future.

## Conclusions

In summary, our results suggest that ACF for TB, both short-term and sustained, may have important impact and are likely to be highly cost-effective within 10 years, even for campaigns costing $1,000 or more per case detected and linked to care. Since most gains in incidence are realized in subsequent years, evaluations over a shorter time span may grossly underestimate the full benefits of ACF. Both longer-term follow-up of existing campaigns and rapid evaluations of highly intensive interventions are needed to fully assess the potential of active TB case finding to avert TB incidence and mortality. In the interim, our “best available evidence” estimates suggest that, if we are to undertake a serious effort to meet TB control targets by 2035, active TB case finding deserves a prominent place on the global health agenda.

## Additional file

Additional file 1:
**Supplemental Methods and Results.**

## Abbreviations

ACF: Active case finding
ART: Antiretroviral therapy
DALY: Disability-adjusted life year
GDP: Gross domestic product
ICER: Incremental cost-effectiveness ratio
TB: Tuberculosis
UR: Uncertainty range
WHO: World Health Organization
