# Supplementary material for: How much is tuberculosis screening worth? Estimating the value of active case finding for tuberculosis in South Africa, China, and India
Source: BMC Med. 2014 Oct 30;12:216. doi: 10.1186/s12916-014-0216-0 (PMC4224697; doi:10.1186/s12916-014-0216-0)
Supplement: Additional file 1: — Supplemental Methods and Results. [file 12916_2014_216_MOESM1_ESM.pdf]

How much is TB Screening Worth? Estimating the Value of Active Case Finding for Tuberculosis in South Africa, China, and India.
Supporting Information

Andrew S. Azman, Jonathan E. Golub, David W. Dowdy

Section S1 Model Description

We used a model of TB transmission that can be represented as a system of ordinary differential equations. The model consists of 9 TB compartments: S, susceptibles; Lf, latently infected (recent); Ls, latently infected (remote); Ps, pre-symptomatic TB infection; Asp, smear-positive TB; Asn, smear-negative pulmonary TB; Aep, extra-pulmonary TB; Tx, on treatment; Rtx, recently treated. Each of these compartments are further sub-divided by HIV status (shown as subscript) with $[-]_0$ representing HIV negative (e.g. S_0 represents TB susceptible that are HIV negative), $[-]_1$ HIV positive CD4 > 350, $[-]_2$ HIV positive CD4 ≤ 350 and not on ART, and $[-]_3$ HIV positive on ART. The values of all parameters used in the primary analysis are shown in Table S1. Each parameter is assumed to be a function of the HIV class they are acting upon; however, for simplicity, in most cases we leave off explicit references to the HIV class. The following text describes the system in detail along with key assumptions of the model. Although this is not an individual-based model, for clarity, we refer to 'individuals' in compartments throughout the description.

The system of equations for HIV-uninfected (group 0) are:

$$\frac{dS_0}{dt} = -S_0(\lambda + \lambda_{hiv}) \tag{1}$$

$$\frac{dLf_0}{dt} = -Lf_0(\gamma_{lf.ls} + \rho_{lf} + \lambda_{hiv}) + \lambda(Ls_0 \cdot \phi_l + Rtx_0 \cdot \phi_l + S_0) \tag{2}$$

$$\frac{dLs_0}{dt} = -Ls_0(\lambda \cdot \phi_l + \rho_{ls} + \lambda_{hiv}) + Lf_0 \cdot \gamma_{lf.ls} + Rtx_0 \cdot \gamma_{rtx.ls} \tag{3}$$

$$\frac{dPs_0}{dt} = -Ps_0(\rho_{ps} + \zeta_{sn} + \lambda_{hiv}) + Lf_0\rho_{lf} + Ls_0\rho_{ls} \tag{4}$$

$$\frac{dAsp_0}{dt} = -Asp_0(\mu_{sp} + \theta + \zeta_{sp} + \lambda_{hiv}) + (Ps_0 \cdot \rho_{ps} + Rtx_0 \cdot \rho_{rel})\pi_{sp}(1 - \pi_{ep}) \tag{5}$$

$$\frac{dAsn_0}{dt} = -Asn_0(\mu_{sn} + \theta + \zeta_{sn} + \lambda_{hiv}) + (Ps_0 \cdot \rho_{ps} + Rtx_0 \cdot \rho_{rel})(1 - \pi_{sp})(1 - \pi_{ep}) \tag{6}$$

$$\frac{dAep_0}{dt} = -Aep_0(\mu_{ep} + \theta + \zeta_{sn} + \lambda_{hiv}) + (Ps_0 \cdot \rho_{ps} + Rtx_0 \cdot \rho_{rel})\pi_{ep} \tag{7}$$

$$\frac{dTx_0}{dt} = -Tx_0(\gamma_{tx.rtx} + \lambda_{hiv}) + \theta(Asp_0 + Asn_0 + Aep_0) \tag{8}$$

$$\frac{dRtx_0}{dt} = -Rtx_0(\gamma_{rtx.ls} + \rho_{rel} + \lambda \cdot \phi_l + \lambda_{hiv}) + Asp_0 \cdot \zeta_{sp} + (Asn_0 + Aep_0 + Ps_0)\zeta_{sn} + Tx_0 \cdot \gamma_{tx.rtx} \tag{9}$$

The system of equations for HIV-infected, CD4 > 350 (group 1) are:

$$\frac{dS_1}{dt} = -S_1(\lambda + \chi_{elg} + \mu_{hiv,1}) + S_0\lambda_{hiv} \tag{10}$$

$$\frac{dLf_1}{dt} = -Lf_1(\gamma_{lf.ls} + \rho_{lf} + \chi_{elg} + \mu_{hiv,1}) + \lambda(Ls_1 \cdot \phi_l + Rtx_1 \cdot \phi_l + S_1) + Lf_0\lambda_{hiv} \tag{11}$$

$$\frac{dLs_1}{dt} = -Ls_1(\lambda \cdot \phi_l + \rho_{ls} + \chi_{elg} + \mu_{hiv,1}) + Lf_1 \cdot \gamma_{lf.ls} + Rtx_1 \cdot \gamma_{rtx.ls} + Ls_0\lambda_{hiv} \tag{12}$$

$$\frac{dPs_1}{dt} = -Ps_1(\rho_{ps} + \zeta_{sn} + \chi_{elg} + \mu_{hiv,1}) + Lf_1\rho_{lf} + Ls_1\rho_{ls} + Ps_0\lambda_{hiv} \tag{13}$$

$$\frac{dAsp_1}{dt} = -Asp_1(\mu_{sp} + \theta + \zeta_{sp} + \chi_{elg} + \mu_{hiv,1}) + (Ps_1 \cdot \rho_{ps} + Rtx_1 \cdot \rho_{rel})\pi_{sp}(1 - \pi_{ep}) + Asp_0\lambda_{hiv} \tag{14}$$

$$\frac{dAsn_1}{dt} = -Asn_1(\mu_{sn} + \theta + \zeta_{sn} + \chi_{elg} + \mu_{hiv,1}) + (Ps_1 \cdot \rho_{ps} + Rtx_1 \cdot \rho_{rel})(1 - \pi_{sp})(1 - \pi_{ep}) + Asn_0\lambda_{hiv} \tag{15}$$

$$\frac{dAep_1}{dt} = -Aep_1(\mu_{ep} + \theta + \zeta_{sn} + \chi_{elg} + \mu_{hiv,1}) + (Ps_1 \cdot \rho_{ps} + Rtx_1 \cdot \rho_{rel})\pi_{ep} + Aep_0\lambda_{hiv} \tag{16}$$

$$\frac{dTx_1}{dt} = -Tx_1(\gamma_{tx.rtx} + \chi_{elg} + \mu_{hiv,1}) + \theta(Asp_1 + Asn_1 + Aep_1) + Tx_0\lambda_{hiv} \tag{17}$$

$$\frac{dRtx_1}{dt} = -Rtx_1(\gamma_{rtx.ls} + \rho_{rel} + \lambda \cdot \phi_l + \chi_{elg} + \mu_{hiv,1}) + Asp_1 \cdot \zeta_{sp} + (Asn_1 + Aep_1 + Ps_1)\zeta_{sn} + Tx_1 \cdot \gamma_{tx.rtx} + Rtx_0\lambda_{hiv} \tag{18}$$

The system of equations for HIV-infected, CD4 ≤ 350 and not on ART (group 2) are:

$$\frac{dS_2}{dt} = -S_2(\lambda + \chi_{tx} + \mu_{hiv,2}) + S_1\chi_{elg} \tag{19}$$

$$\frac{dLf_2}{dt} = -Lf_2(\gamma_{lf.ls} + \rho_{lf} + \chi_{tx} + \mu_{hiv,2}) + \lambda(Ls_2 \cdot \phi_l + Rtx_2 \cdot \phi_l) + Lf_1\chi_{elg} \tag{20}$$

$$\frac{dLs_2}{dt} = -Ls_2(\lambda \cdot \phi_l + \rho_{ls} + \chi_{tx} + \mu_{hiv,2}) + Lf_2 \cdot \gamma_{lf.ls} + Rtx_2 \cdot \gamma_{rtx.ls} + Ls_1\chi_{elg} \tag{21}$$

$$\frac{dPs_2}{dt} = -Ps_2(\rho_{ps} + \zeta_{sn} + \chi_{tx} + \mu_{hiv,2}) + Lf_2\rho_{lf} + Ls_2\rho_{ls} + Ps_1\chi_{elg} \tag{22}$$

$$\frac{dAsp_2}{dt} = -Asp_2(\mu_{sp} + \theta + \zeta_{sp} + \chi_{tx} + \mu_{hiv,2}) + (Ps_2 \cdot \rho_{ps} + Rtx_2 \cdot \rho_{rel})\pi_{sp}(1 - \pi_{ep}) + Asp_1\chi_{elg} \tag{23}$$

$$\frac{dAsn_2}{dt} = -Asn_2(\mu_{sn} + \theta + \zeta_{sn} + \chi_{tx} + \mu_{hiv,2}) + (Ps_2 \cdot \rho_{ps} + Rtx_2 \cdot \rho_{rel})(1 - \pi_{sp})(1 - \pi_{ep}) + Asn_1\chi_{elg} \tag{24}$$

$$\frac{dAep_2}{dt} = -Aep_2(\mu_{ep} + \theta + \zeta_{sn} + \chi_{tx} + \mu_{hiv,2}) + (Ps_2 \cdot \rho_{ps} + Rtx_2 \cdot \rho_{rel})\pi_{ep} + Aep_1\chi_{elg} \tag{25}$$

$$\frac{dTx_2}{dt} = -Tx_2(\gamma_{tx.rtx} + \chi_{tx} + \mu_{hiv,2}) + \theta(Asp_2 + Asn_2 + Aep_2) + Tx_1\chi_{elg} \tag{26}$$

$$\frac{dRtx_2}{dt} = -Rtx_2(\gamma_{rtx.ls} + \rho_{rel} + \lambda \cdot \phi_l + \chi_{tx} + \mu_{hiv,2}) + Asp_2 \cdot \zeta_{sp} + (Asn_2 + Aep_2 + Ps_2)\zeta_{sn} + Tx_2 \cdot \gamma_{tx.rtx} + Rtx_1\chi_{elg} \tag{27}$$

The system of equations for HIV-infected on ART (group 3) are:

$$\frac{dS_3}{dt} = -S_3(\lambda + \mu_{hiv,3}) + S_2\chi_{tx} \quad (28)$$

$$\frac{dLf_3}{dt} = -Lf_3(\gamma_{lf.ls} + \rho_{lf} + \mu_{hiv,3}) + \lambda(Ls_3 \cdot \phi_l + Rtx_3 \cdot \phi_l) + Lf_2\chi_{tx} \quad (29)$$

$$\frac{dLs_3}{dt} = -Ls_3(\lambda \cdot \phi_l + \rho_{ls} + \mu_{hiv,3}) + Lf_3 \cdot \gamma_{lf.ls} + Rtx_3 \cdot \gamma_{rtx.ls} + Ls_2\chi_{tx} \quad (30)$$

$$\frac{dPs_3}{dt} = -Ps_3(\rho_{ps} + \zeta_{sn} + \mu_{hiv,3}) + Lf_3\rho_{lf} + Ls_3\rho_{ls} + Ps_2\chi_{tx} \quad (31)$$

$$\frac{dAsp_3}{dt} = -Asp_3(\mu_{sp} + \theta + \zeta_{sp} + \mu_{hiv,3}) + (Ps_3 \cdot \rho_{ps} + Rtx_3 \cdot \rho_{rel})\pi_{sp}(1 - \pi_{ep}) + Asp_2\chi_{tx} \quad (32)$$

$$\frac{dAsn_3}{dt} = -Asn_3(\mu_{sn} + \theta + \zeta_{sn} + \mu_{hiv,3}) + (Ps_3 \cdot \rho_{ps} + Rtx_3 \cdot \rho_{rel})(1 - \pi_{sp})(1 - \pi_{ep}) + Asn_2\chi_{tx} \quad (33)$$

$$\frac{dAep_3}{dt} = -Aep_3(\mu_{ep} + \theta + \zeta_{sn} + \mu_{hiv,3}) + (Ps_3 \cdot \rho_{ps} + Rtx_3 \cdot \rho_{rel})\pi_{ep} + Aep_2\chi_{tx} \quad (34)$$

$$\frac{dTx_3}{dt} = -Tx_3(\gamma_{tx.rtx} + \mu_{hiv,3}) + \theta(Asp_3 + Asn_3 + Aep_3) + Tx_2\chi_{tx} \quad (35)$$

$$\frac{dRtx_3}{dt} = -Rtx_3(\gamma_{rtx.ls} + \rho_{rel} + \lambda \cdot \phi_l + \mu_{hiv,3}) + Asp_3 \cdot \zeta_{sp} + (Asn_3 + Aep_3 + Ps_3)\zeta_{sn} + Tx_3 \cdot \gamma_{tx.rtx} + Rtx_2\chi_{tx} \quad (36)$$

Susceptible - S: Equations 1, 10, 19, and 28 describe the rates in and out of the susceptible classes. Susceptibles become infected with TB at a rate of λ given by:

$$\lambda = \frac{\beta_{sp}}{N} \sum_{i \in \{0,1,2,3\}} \left(Asp_i + (Asn_i + Ps_i) \cdot \phi_{sn}\right), \quad (37)$$

where N represents the total population size. We assumed that smear-negative pulmonary TB is a fraction, ϕ_{sp}, of the infectiousness of smear-positive TB, and that extra-pulmonary TB is non-infectious. To keep a constant population size, all deaths from disease (TB and HIV) were replaced with HIV-negative susceptibles (not shown in equations for simplicity).

Latent, recent - Lf: Equations 2, 11, 20, 29 show the rates of transition in and out of the latent (recent) compartments. New individuals enter the recently infected latent class through; (1) new infections via the susceptible class (λS), (2) reinfections from recently treated individuals with partial immunity ($\lambda \cdot Rtx \cdot \phi_l$), or (3) reinfections from partially immune individuals with remote latent infections ($\lambda \cdot Ls \cdot \phi_l$). Individuals leave this compartment either by progressing to pre-symptomatic TB at a rate of ρ_{lf}, or by progressing to remote latent infection at a rate of $\gamma_{lf.ls}$.

Latent, remote - Ls: Equations 3, 12, 21, and 30 show the rates of transition in and out of the latent (remote) compartments. New individuals enter the remotely infected latent class through progression from a recent latent infection ($Ls \cdot \gamma_{ltx.ls}$), or through stabilization after successful treatment or self-cure, at a rate of $Rtx \cdot \gamma_{rtx.ls}$. Individuals leave this compartment for the pre-symptomatic compartment at a rate of ρ_{ls}, the inverse of the duration of the 'recently infected' compartment.

Pre-symptomatic, Ps: Equations 4, 13, 22, and 31 show the rates of transition in and out of the pre-symptomatic TB class. Individuals in this class are assumed to be less infectious than fully-symptomatic smear-positive cases (ϕ_{sn} times less). Individuals move into this compartment by progressing from recent or remote latency (Lf or Ls). They leave this class by either spontaneous self-cure at a rate of ζ_{sn} (assumed to happen at the same rate as it does for smear-negative pulmonary TB), or by progressing to active TB at a rate of ρ_{ps}. We assume that there is no TB associated mortality at this stage of disease.

Active TB, Asp/Asn/Aep: Equations 5, 14, 23, 32, 6, 15, 24, 33, 7, 16, 25, and 34 show the rates of transition for smear-positive (Asp), smear-negative pulmonary (Asn), and extra-pulmonary (Aep) TB. Individuals progress to active TB either from the pre-symptomatic phase at a rate of $Ps \cdot \rho_{ps}$, or by relapsing after recent TB treatment at a rate of $Rtx \cdot \rho_{rel}$. The proportion of individuals progressing to pulmonary TB is $1 - \pi_{ep}$, and π_{sp} represents the proportion of pulmonary TB cases that are smear-positive. Individuals leave the Active TB compartments through TB associated death at a rate of $\mu_{sp/sn/ep}$, by spontaneous cure at a rate of $\zeta_{sp/sn/ep}$, or through detection and treatment at a rate of θ.

Treatment, Tx: Equations 8, 17, 26, and 35 show the rates in and out of compartments representing those on TB treatment. Those with active TB enter this compartment at a rate of $A(sp/sn/ep) \cdot \theta$ and exit the compartment on average, 6-months later (a rate of $\gamma_{tx.rtx}$). No re-infections can take place during this time and we assume that all deaths

on TB treatment reflect mortality risk accrued during the active TB phases with no additional risk of TB associated death in these compartments.

Recently Treated/Recovered, Rtx: Equation 9, 18, 27, and 36 show the rates of transition in and out of the recently treated/recovered compartments. Individuals enter these compartments from treatment at a rate of $\gamma_{tx.rtx}$, and through spontaneous cure from pre-symptomatic (at a rate of $Ps \cdot \zeta_{sn}$) or active TB (at a rate of $Asp/sn/ep \cdot \zeta_{sp/sn/ep}$). From this compartment, individuals can either be reinfected ($\lambda\phi_l$), relapse (ρ_{rel}), or progress to remote latent infection ($\gamma_{rtx.ls}$) an average of 5 years later.

HIV Compartments: To account for HIV, we created parallel compartments (Figure S1) representing HIV^- (0), HIV^+ $CD4 \geq 350$ (1), HIV^+ $CD4 < 350$ not on ART (2), and HIV^+ on ART (3). Individuals in the HIV^- compartments were assumed to move at a constant rate, λ_{hiv}, to the HIV^+ $CD4 \geq 350$ compartments (from the circles shown in white to those in yellow in Figure S1). Based on data on the natural history of HIV,[1] those inHIV^+ $CD4 \geq 350$ progressed to HIV^+ $CD4 < 350$ not on ART at a constant rate χ_{elg}. Those in HIV^+ $CD4 < 350$ not on ART then go on HIV treatment at a constant rate of χ_{tx}. All HIV^+ classes $(1, 2, \text{and } 3)$ are assumed to have an increased mortality rate, $\mu_{hiv,\cdot}$. When insufficient data was available to assign specific parameter values to all HIV sub-compartments, we assumed that those corresponding to individuals on ART and those with $CD4 > 350$ were a weighted average of HIV^- and $CD4 < 350$ not on ART parameters with the weight, κ (0.7), on class HIV^- and $(1 - \kappa)$ on class $CD4 < 350$ not on ART.

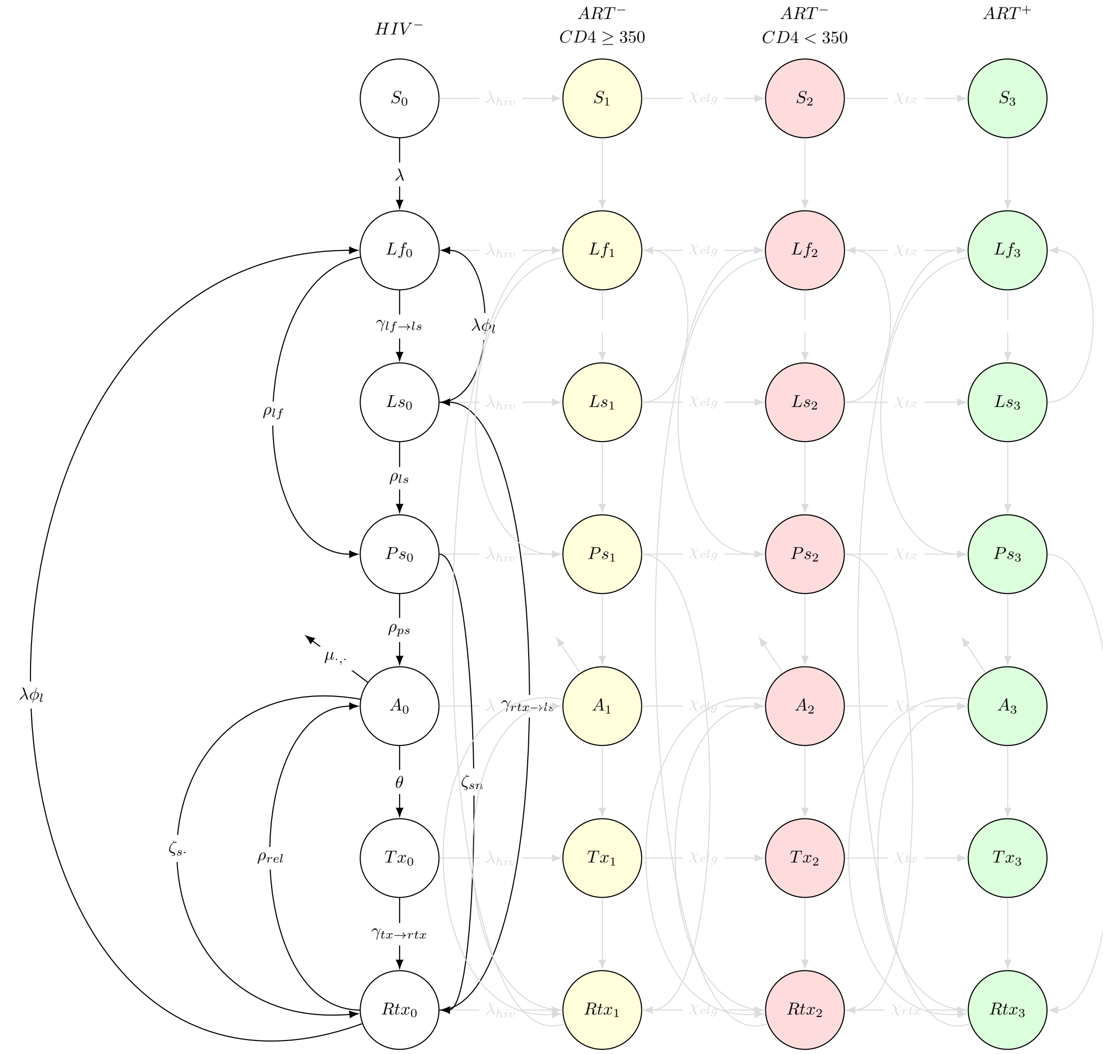

Figure S1: HIV-TB model structure. The main TB model is shown on the left most column in white. Rates between each compartment are shown on the lines with the arrow heads indicating the flow direction. The arrows representing flows between HIV states are shown in grey, along with a set of unlabeled arrows for the TB flows within the HIV^+ states.

Section S2 Transmission Parameters

Table S1: TB and HIV parameters used for models in each country by HIV class (columns). All rates are expressed in units of yr^{-1} unless otherwise noted. When three values are shown in a single cell (e.g. the first row), they represent values from India/China/South Africa respectively.

		0	1	2	3	Ref
β_{sp}	Number of TB infections per smear-positive case	16.16/ 11.16/ 23.63	16.16/ 11.16/ 23.63	16.16/ 11.16/ 23.63	16.16/ 11.16/ 23.63	fit
ϕ_{sn}	Relative transmissibility of smear-negative TB	0.22	0.22	0.22	0.22	[2]
ϕ_{ps}	Relative transmissibility of pre-symptomatic TB	0.22	0.22	0.22	0.22	assumed
$\gamma_{lf.ls}$	Rate of stabilization from early to late latency	0.50	0.50	0.50	0.50	[3]
$\gamma_{rtx.ls}$	Rate of stabilization after successful treatment	0.20	0.20	0.20	0.20	[3]
$\gamma_{tx.rtx}$	Rate of treatment	2.00	2.00	2.00	2.00	[4]
ϕ_l	Relative risk of reinfection	0.21	0.45	1.00	0.45	[5, 6]
ρ_{lf}	Rate of rapid progression to active TB after recent infection	0.07	0.26	0.70	0.26	[3]
ρ_{ls}	Rate of endogenous reactivation to active TB after remote infection	$4.9 \times 10^{-}$	0.024	0.08	0.024	[7, 8]
ρ_{rel}	Relapse rate from active TB	0.02	0.02	0.02	0.02	[6]
ρ_{ps}	Rate of development of active TB from pre-symptomatic stage	1.33	3.93	10.00	3.93	assumed
π_{ep}	Proportion of TB that is extra-pulmonary	0.15	0.23	0.40	0.23	assumed
π_{sp}	Proportion of pulmonary TB that is smear-positive	0.75	0.65	0.40	0.65	[4]
μ_{sp}	Mortality rate from smear-positive TB	0.23	0.56	1.33	0.56	[9]
μ_{sn}	Mortality rate from smear-negative pulmonary TB	0.18	0.53	1.33	0.53	[9]
μ_{ep}	Mortality rate from extra pulmonary TB	0.18	0.53	1.33	0.53	[9]
ζ_{sp}	Self cure rate, smear-positive	0.10	0.07	0.00	0.07	[9]
ζ_{sn}	Self cure rate, smear-negative/extra pulmonary	0.15	0.10	0.00	0.10	[9]
θ	Rate of detection and diagnosis	1.01/ 1.05/ 1.94	1.01/ 1.05/ 1.94	1.01/ 1.05/ 1.94	1.01/ 1.05/ 1.94	fit
λ_{hiv}	Rate of new HIV infections (per 10,000 person-yrs)	3.4/ 0.004/ 212.4	0.00	0.00	0.00	fit
χ_{elg}	Rate of CD4 decline from infection to CD4=350	0.00	0.24	0.00	0.00	[1]
χ_{tx}	Rate of progression from ART eligibility to on ART	0.00	0.00	0.04/ 0.06/ 0.19	0.00	fit
μ_{hiv}	Excess mortality rate from HIV	0.00	0.01	0.13	0.04	[10]
δ	Background mortality rate	0.02	0.02	0.02	0.02	assumed
κ	HIV weight (see Model Description)	0.70	0.70	0.70	0.70	assumed

Section S3 Model Calibration

The models for each country were calibrated to reflect key attributes of the TB and HIV epidemics. We adjusted β_{sp} and θ_{sp} to match each country's TB incidence and case detection proportion (sometimes referred to as the case detection rate) as estimated by the WHO. The HIV parameters λ_{hiv} and χ_{tx} were adjusted to yield each country's HIV incidence and proportion of eligible adult HIV^+ individuals on ART (HIV class 3).

Since the TB incidence in both India and China has consistently declined in the years leading up to 2011, we fit models for both of these countries to data from 2004 [4]. We then found the (constant) rate of decline in β_{sp} necessary to have the epidemic match the TB and HIV data for 2011. In South Africa, the TB incidence has been nearly constant (according to [4]) so we directly calibrated the model to a steady-state 2011. Key outputs from the each of the models are shown in the Table S2.

Table S2: Key epidemiologic outputs (per 100,000 population) from models in years calibrated to data from [4, 11, 12]. Note that all these represent the figures for adults only.

	TB Mort. HIV^- only	TB Mort. HIV^+ only	TB Prev. HIV^+ only	TB Prev.	TB Inc.	TB Inc. HIV^+ only	HIV Prev.	% on ART
India - 2004	28.70	4.80	9.90	333.90	212.00	11.10	4.0×10^{-4}	40
India - 2011	25.70	4.50	9.30	291.30	181.00	10.50	4.0×10^{-4}	40
China - 2004	13.00	<1e-6	<1e-6	150.90	95.00	<1e-6	4.6×10^{-6}	50
China - 2011	11.10	<1e-6	<1e-6	123.30	75.00	<1e-6	4.6×10^{-6}	50
South Africa - 2011	18.70	206.60	513.80	877.00	993.20	714.30	0.17	75

Section S4 Active Case Finding Intervention Size

We modeled the ACF intervention in main manuscript as a 25% increase in the number of cases (Asp, Asn, and Aep) that would have been found in the first year in the absence of ACF (through increasing the rate of diagnosis and detection, θ). For example, we estimate 124 cases per 100,000 would be found in India in 2012; therefore, we assume the intervention will detect an additional $124 \times 0.25 = 31$ cases in the first year. We based this number roughly on the percentage of all smear positive cases detected by the Enhanced Case Finding (ECF) arm of the ZAMSTAR trial [13]. Although many of the individuals detected by ECF may otherwise have been detected by the passive system, such that 29% represents an upper bound of the effect achieved through enhanced case-finding within ZAMSTAR, we use this benchmark as being an ambitious yet feasible target for a TB case-finding program, rather than arguing that the ZAMSTAR intervention actually achieved this level of effect. Figure S2 illustrates how the estimated cost-effectiveness threshold changes as the size of the ACF campaign is changed.

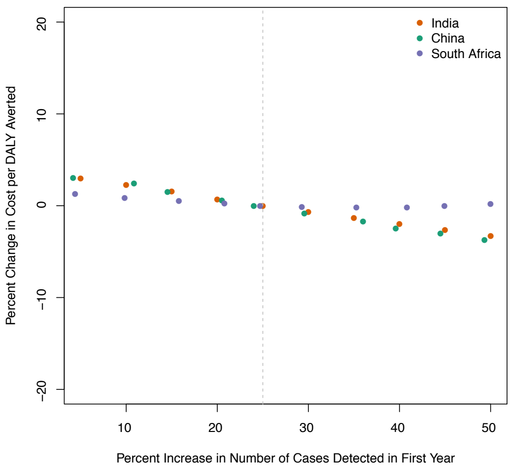

Figure S2: Effect of ACF intensity on cost per DALY averted. Percent change in cost per DALY (y-axis) for ACF interventions ($1500 per case detected) of different intensity and shown on the y-axis as the percent increase in case detection in the first year. Note that the points positions along the x-axis are slightly jittered for illustrative clarity. Vertical line indicates ACF intensity used in main analysis.

As seen in Figure S2, the relationship between *cost per additional case diagnosed and treated* and *cost per DALY*

averted is robust to the size of the ACF campaign. In many cases, there may be efficiencies of scale that reduce the cost per additional case diagnosed and treated in larger campaigns. In such cases, the *cost per DALY averted* would be smaller for a larger campaign, but that cost could still be estimated from Figures 2 and 4 (i.e., the larger campaign would fall at a different place on the cost-per-case-diagnosed axis).

Section S5 Economic Analyses

To calculate the cost per DALY, we used outputs from the transmission model along with a number of cost-related assumptions. We allowed benefits and costs to accrue only up to the specified analytic horizon. For example, if the analytic horizon were 3 years, deaths averted in the 4th year (or anytime afterwards) as a result of the intervention would not be included in any estimates of the cost-effectiveness. All future costs and benefits were discounted at 3%. To calculate the incremental DALYs averted, we computed the years of life lost (YLL, Equation 38) and years of life with disability (YLD, Equation 39) for both the counterfactual and active case finding scenarios. The equations for both are as follows:

$$YLL = \int_{t=0}^{H} \int_{u=0}^{H-t} e^{-0.03t} \left(\sum_{j \in hiv\ group} \mu_{sp,j} Asp_j(t) + \mu_{sn,j} Asn_j(t) + \mu_{ep,j} Aep_j(t) \right) dudt \quad (38)$$

$$YLD = \int_{t} \sum_{j \in hiv\ group} Tx_j(t) w_{Tx,j} + w_{TB,j} \left[Asp_j(t) + Asn_j(t) + Aep_j(t) \right] \ dt \quad (39)$$

where H is the analytic horizon. We then subtracted the estimates from both scenarios and took their sum so that:

$$\text{incremental DALYs averted} = (YLL_{counterfactual} - YLL_{ACF}) + (YLD_{counterfactual} - YLD_{ACF}) \quad (40)$$

We calculate YLD for each group by Equation 39 where $w_{TB,j}$ represents the disability weight assigned to TB infections by HIV status ($j \in \{0, 1, 2, 3\}$), and $w_{Tx,j}$ represents the disability weight for time on TB treatment. The disability weights for TB disease, adapted from Salomon et al [14] are 0.331, 0.399, 0.547, 0.399, for HIV groups 0-3, respectively. The disability weights for TB treatment, 0.166, 0.310, 0.547, 0.226, are assumed to be the average of the weight for TB disease with or without HIV co-infection and the weight for HIV infection with or without ARTs (when HIV^{+}).

We calculate the incremental cost difference by subtracting the treatment and detection/diagnosis costs for the baseline scenario from that of the active case finding scenario. Treatment costs were approximated as the treatment costs for standard first-line regimens, as reported estimated by the WHO [4], times the proportion of incident cases estimated to be drug-susceptible plus the second-line drug cost (assumed to be $4672 in South Africa, and $4396 in China, and $2560 in India [4]) times the proportion of incident cases estimated to be MDR TB (1.8% in South Africa, 2.1% in India, and 5.7% in China [4]).

Section S6 Additional Results Figures

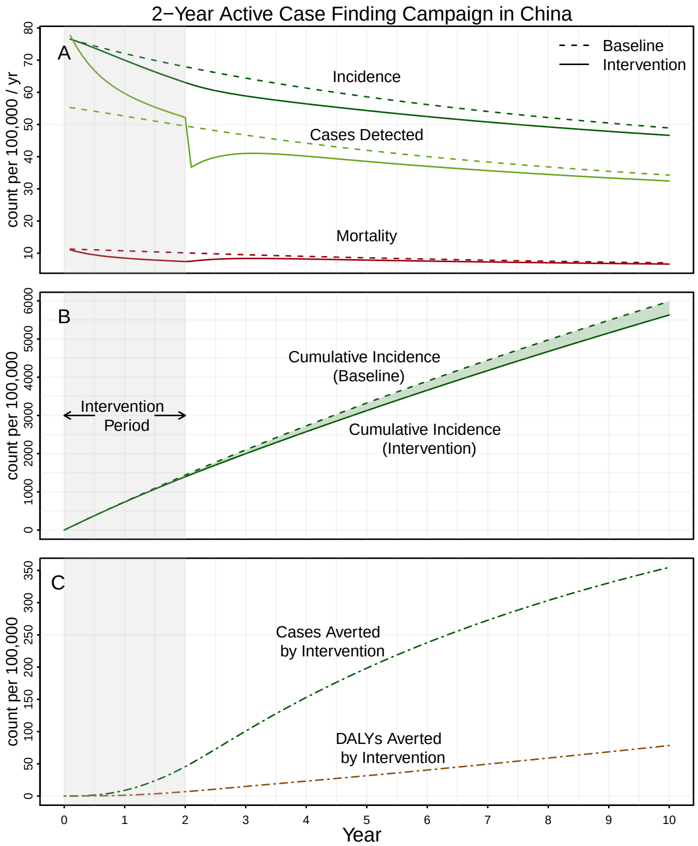

Figure S3: Impact of a discrete 2-Year active case finding campaign in China. Panel A illustrates the incidence (dark green), case detection rate (light green), and mortality rate (red) for a baseline/counterfactual scenario (dashed) compared against an intervention scenario (solid) in which TB case detection is increased, through active case finding, by 25% from the cases detected in the first year (2012). Panel B shows the cumulative incidence (per 100,000) for both the intervention (solid) and baseline (dashed) scenarios with the area between the two curves representing the cases averted through active case finding. Panel C shows the cases averted by the intervention (green), and DALYs averted by the intervention (brown) a function of cases averted and mortality averted by intervention. The grey shading highlights the component of the intervention effect that would be observable during the course of a two-year intervention study.

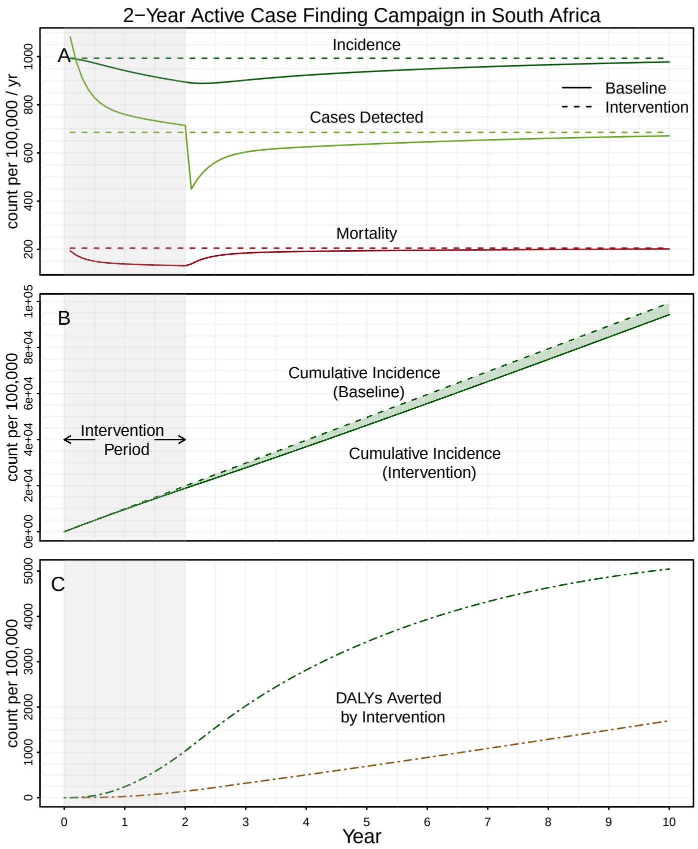

Figure S4: Impact of a discrete 2-Year active case finding campaign in South Africa. Panel A illustrates the incidence (dark green), case detection rate (light green), and mortality rate (red) for a baseline/counterfactual scenario (dashed) compared against an intervention scenario (solid) in which TB case detection is increased, through active case finding, by 25% from the cases detected in the first year (2012). Panel B shows the cumulative incidence (per 100,000) for both the intervention (solid) and baseline (dashed) scenarios with the area between the two curves representing the cases averted through active case finding. Panel C shows the cases averted by the intervention (green), and DALYs averted by the intervention (brown) a function of cases averted and mortality averted by intervention. The grey shading highlights the component of the intervention effect that would be observable during the course of a two-year intervention study.

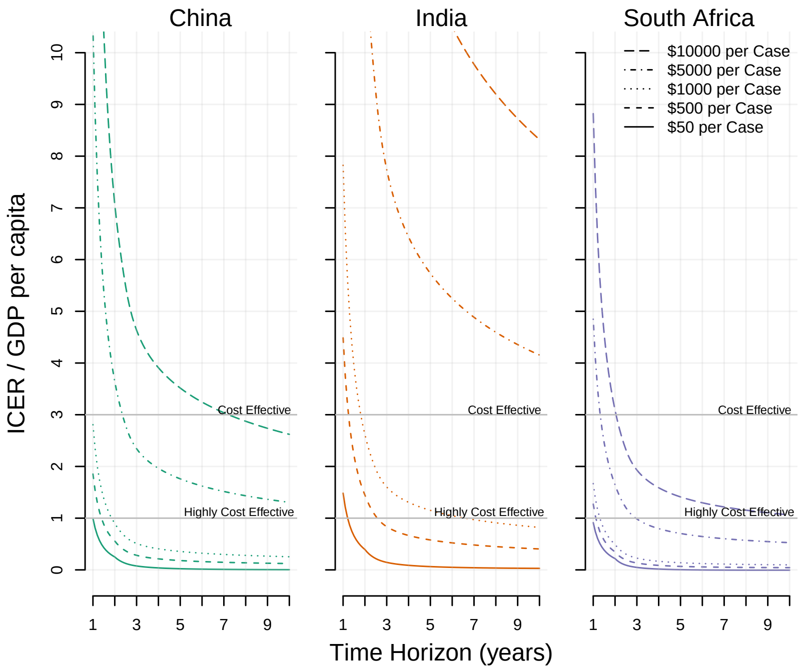

Figure S5: Cost effectiveness thresholds per country by cost per case and analytic horizon. The lines show the ratio of the estimated ICER for a 2 year campaign divided by the GDP of each country (columns) with each line type representing a different cost per case detected. The two grey horizontal lines on each represent the thresholds commonly used to define cost effectiveness of health interventions.[15]

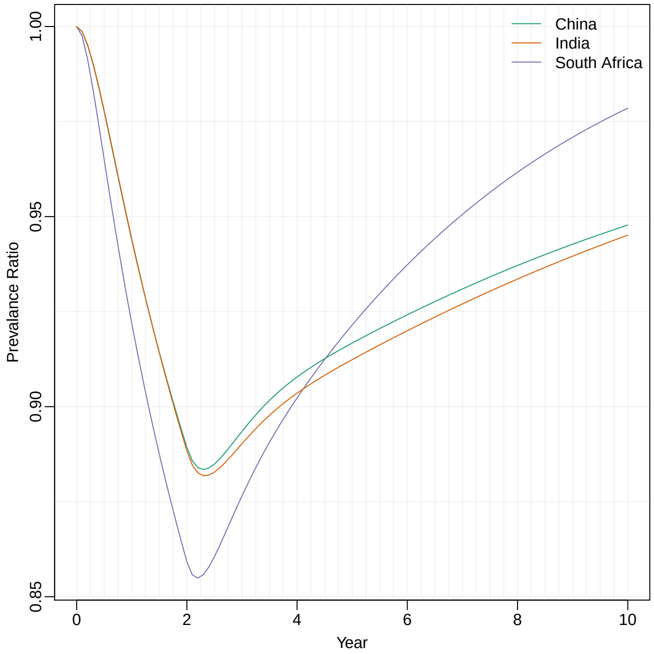

Figure S6: Prevalence ratio of TB, comparing a 2-year ACF campaign to the counterfactual. Prevalence ratio of TB is calculated as the point prevalence of the intervention scenario divided by the point prevalence of the counterfactual scenario.

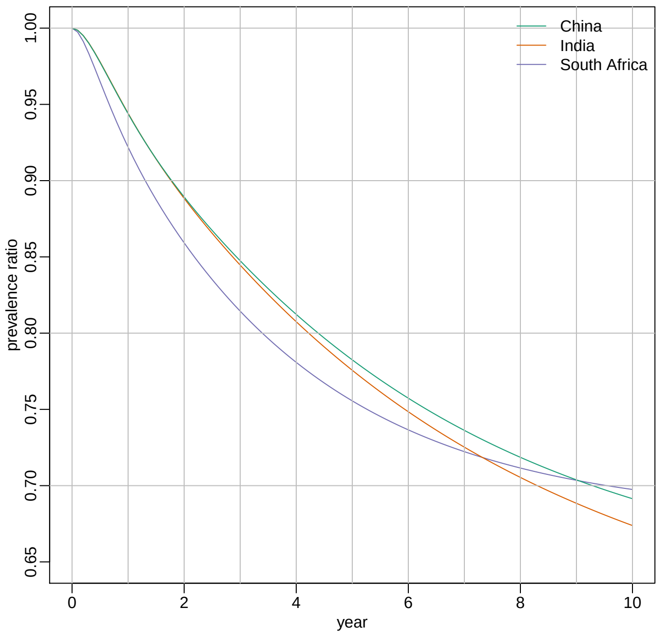

Figure S7: Prevalence ratio of TB, comparing a 10-year ACF campaign to the counterfactual. Prevalence ratio of TB is calculated as the point prevalence of the intervention scenario divided by the point prevalence of the counterfactual scenario.

Section S7 Sensitivity & Uncertainty Analyses

Section S7.1 One Way Sensitivity Analyses

In one-way sensitivity analyses, we varied each parameter by 50% of the value used in the main analysis (Table S1), then starting the model at the same 2011 initial conditions as the main analyses, ran the model and then calculated the cost per DALY averted. The results for India, China, and South Africa are shown in Figures S8, S10, and S9.

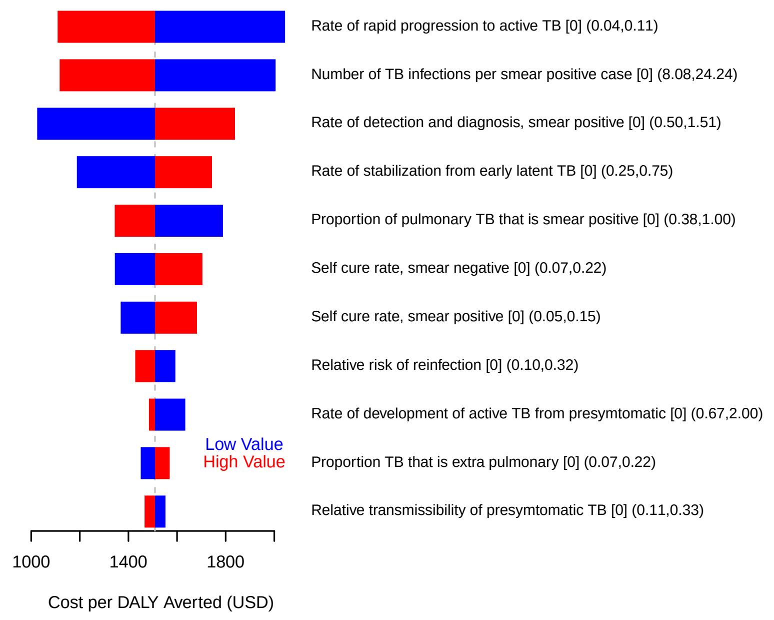

Figure S8: One-way sensitivity analysis for India using a 10-year analytic horizon at a cost of $1200 per case detected. This plot shows the estimated costs per DALY averted of a discrete two-year campaign, with costs and effects evaluated over a ten-year horizon, for India assuming the cost per case detected is $1,200. The number next to each parameter (e.g. [0]) indicates the HIV class (see Figure S1). Red bars represent high values of the parameters and blue bars represent low values of the parameters. The 11 parameters with the widest uncertainty range are shown. The range of each parameter went from ±50% of the estimated value (or the maximum/minimum possible value in the case of bounded parameters, e.g. proportions). Note: rate of rapid progression refers to the rate of progression per year from the recently infected, latent compartment - which lasts a mean of 2 years.

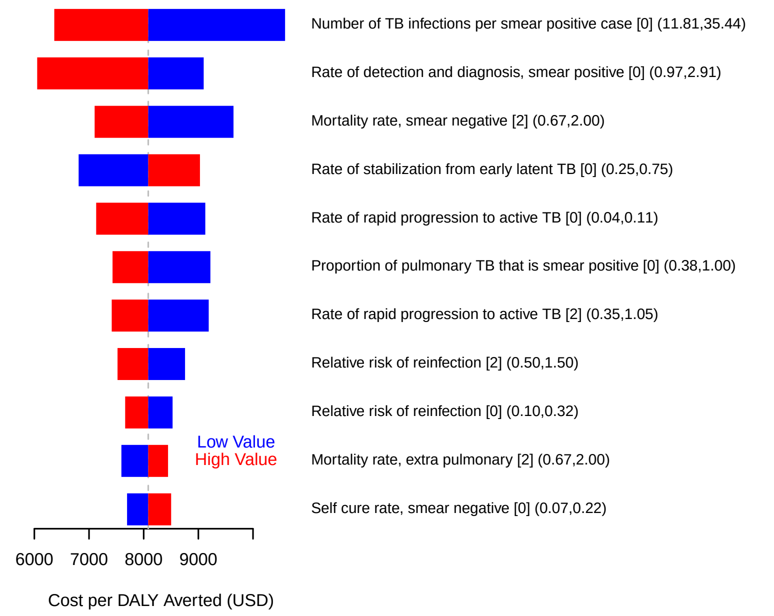

Figure S9: One-way sensitivity analysis for South Africa using a 10-year analytic horizon at a cost of $9,400 per case detected. This plot shows the estimated costs per DALY averted of a discrete two-year campaign, with costs and effects evaluated over a ten-year horizon, for South Africa assuming the cost per case detected is $9,400. The number next to each parameter (e.g. [0]) indicates the HIV class (see Figure S1). Red bars represent high values of the parameters and blue bars represent low values of the parameters. The 10 parameters with the widest uncertainty range are shown. The range of each parameter went from ±50% of the estimated value (or the maximum/minimum possible value in the case of bounded parameters, e.g. proportions). Note: rate of rapid progression refers to the rate of progression per year from the recently infected, latent compartment - which lasts a mean of 2 years.

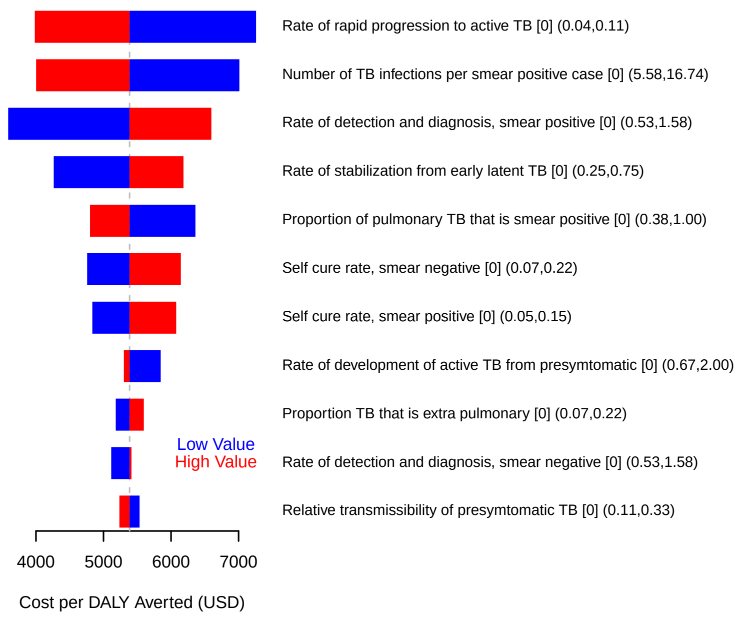

Figure S10: One-way sensitivity analysis for China using a 10-year analytic horizon at a cost of $3800 per case detected. This plot shows the estimated costs per DALY averted of a discrete two-year campaign, with costs and effects evaluated over a ten-year horizon, for China assuming the cost per case detected is $3,800. The number next to each parameter (e.g. [0]) indicates the HIV class (see Figure S1). Red bars represent high values of the parameters and blue bars represent low values of the parameters. The 11 parameters with the widest uncertainty range are shown. The range of each parameter went from ±50% of the estimated value (or the maximum/minimum possible value in the case of bounded parameters, e.g. proportions). Note: rate of rapid progression refers to the rate of progression per year from the recently infected, latent compartment - which lasts a mean of 2 years.

Section S7.2 Multivariate Uncertainty Analyses

Table S3: Ranges explored for parameters in multi-variable uncertainty analyses, by country. Random sets of parameters were drawn, using Latin Hypercube Resampling, from independent beta distributions with a mode, min, and max, as shown in the table and a shape parameter equal to 3.

Parameter	HIV	India Mode	India Min	India Max	China Mode	China Min	China Max	South Africa Mode	South Africa Min	South Africa Max
β_{sp}	0	16.16	8.08	24.24	11.16	5.58	16.74	23.63	11.82	35.45
ϕ_{sn}	0	0.22	0.11	0.33	0.22	0.11	0.33	0.22	0.11	0.33
ϕ_{l}	0	0.21	0.10	0.32	0.21	0.10	0.32	0.21	0.10	0.32
ϕ_{l}	2	1.00	0.50	1.50	1.00	0.50	1.50	1.00	0.50	1.50
ϕ_{ps}	0	0.22	0.11	0.33	0.22	0.11	0.33	0.22	0.11	0.33
$\gamma_{lf.ls}$	0	0.50	0.25	0.75	0.50	0.25	0.75	0.50	0.25	0.75
$\gamma_{rtx.ls}$	0	0.20	0.10	0.30	0.20	0.10	0.30	0.20	0.10	0.30
$\gamma_{tx.rtx}$	0	2.00	1.00	3.00	2.00	1.00	3.00	2.00	1.00	3.00
ρ_{lf}	0	0.07	0.04	0.10	0.07	0.04	0.10	0.07	0.04	0.10
ρ_{lf}	2	0.70	0.35	1.05	0.70	0.35	1.05	0.70	0.35	1.05
ρ_{ls}	0	0.00	0.00	0.00	0.00	0.00	0.00	0.00	0.00	0.00
ρ_{ls}	2	0.02	0.01	0.03	0.02	0.01	0.03	0.02	0.01	0.03
ρ_{rel}	0	0.02	0.01	0.03	0.02	0.01	0.03	0.02	0.01	0.03
ρ_{ps}	0	1.33	0.67	2.00	1.33	0.67	2.00	1.33	0.67	2.00
ρ_{ps}	2	10.00	5.00	15.00	10.00	5.00	15.0	10.00	5.00	15.0
π_{sp}	0	0.75	0.38	1.12	0.75	0.38	1.00	0.75	0.38	1.00
π_{sp}	2	0.40	0.20	0.60	0.40	0.20	0.60	0.40	0.20	0.60
π_{ep}	0	0.15	0.07	0.23	0.15	0.07	0.23	0.15	0.07	0.23
π_{ep}	2	0.40	0.20	0.60	0.40	0.20	0.60	0.40	0.20	0.60
μ_{sp}	2	1.33	0.67	2.00	1.33	0.67	2.00	1.33	0.67	2.00
μ_{sn}	2	1.33	0.67	2.00	1.33	0.67	2.00	1.33	0.67	2.00
μ_{ep}	2	1.33	0.67	2.00	1.33	0.67	2.00	1.33	0.67	2.00
ζ_{sp}	0	0.10	0.05	0.15	0.10	0.05	0.15	0.10	0.05	0.15
ζ_{sp}	2	0.00	0.00	0.10	0.00	0.00	0.15	0.00	0.00	0.15
ζ_{sn}	0	0.15	0.07	0.23	0.15	0.07	0.23	0.15	0.07	0.23
ζ_{sn}	2	0.00	0.00	0.15	0.00	0.00	0.15	0.00	0.00	0.15
ζ_{ep}	0	0.15	0.07	0.23	0.15	0.07	0.23	0.15	0.07	0.23
ζ_{ep}	2	0.00	0.00	0.15	0.00	0.00	0.15	0.00	0.00	0.15
θ	0	1.01	0.50	1.51	1.05	0.53	1.58	1.94	0.97	2.91
λ_{hiv}	0	0.0004	0.0002	0.0005	0.00	0.00	0.00	0.02	0.01	0.03
χ_{elg}	1	0.24	0.12	0.36	0.24	0.12	0.36	0.24	0.12	0.36
χ_{tx}	2	0.04	0.02	0.06	0.06	0.03	0.09	0.19	0.10	0.28
μ_{hiv}	1	0.01	0.00	0.01	0.01	0.00	0.01	0.01	0.00	0.01
μ_{hiv}	2	0.13	0.07	0.20	0.13	0.07	0.20	0.13	0.07	0.20
μ_{hiv}	3	0.04	0.02	0.05	0.04	0.02	0.05	0.04	0.02	0.05
κ	-	0.70	0.35	1.00	0.70	0.35	1.00	0.70	0.35	1.00
1st-line Treatment Cost ($)	-	81.0	40.5	121.5	232.0	116.0	38.0	1029.0	514.5	1543.5
2nd-line Treatment Cost ($)	-	4396	2198	6594	4396	2198	6594	4672	2336	7008

Table S4: Estimates and 95% uncertainty ranges for cost per case detected to yield a highly cost-effective 2-year ACF campaign (i.e. where the cost per DALY averted equals the per capita GDP).

Analytic Horizon	India Est.	India 2.5%	India 97.5%	China Est.	China 2.5%	China 97.5%	South Africa Est.	South Africa 2.5%	South Africa 97.5%
10-year	1215	850	2043	3837	2706	6392	9408	6957	13221
5-year	868	691	1151	2834	2257	3731	7094	5580	9022
2-year	309	275	343	1146	1030	1258	2760	2272	3174

Table S5: Partial Rank Correlation Coefficients for ICER with a 10-year analytic horizon from a 2-year ACF campaign in each country.

	HIV Class	India	China	South Africa
Rate of rapid progression to active TB	0	0.89	0.89	0.70
Number of TB infections per smear-positive	0	0.89	0.88	0.90
Rate of detection and diagnosis, smear-positive	0	-0.87	-0.88	-0.80
Rate of stabilization from early to late latency	0	-0.75	-0.75	-0.72
Proportion of pulmonary TB that is smear-positive	0	0.72	0.64	0.60
Self cure rate, smear-negative	0	-0.63	-0.68	-0.39
Self cure rate, smear-positive	0	-0.54	-0.60	-0.26
Relative risk of reinfection	0	0.33	0.11	0.38
Proportion TB that is extra pulmonary	0	-0.24	-0.24	-0.21
Rate of development of active TB from pre-symptomatic	0	0.23	0.21	-0.10
Relative transmissibility of pre-symptomatic	0	0.19	0.16	0.17
Rate of detection and diagnosis, smear-negative	0	-0.13	-0.17	-0.08
Relative transmissibility, smear-negative	0	0.11	0.12	0.18
Mortality rate from smear-negative TB	2	0.10	-0.01	0.77
Mortality rate from extra pulmonary TB	2	-0.08	0.02	-0.39
Rate of rapid progression to active TB	2	0.08	-0.01	0.62
Rate of endogenous reactivation to active TB	0	0.07	0.12	0.00
Rate of detection and diagnosis, extra pulmonary	0	-0.04	-0.01	0.06
Rate of endogenous reactivation to active TB	2	0.04	0.01	0.03
Rate of new HIV infections	0	0.04	0.02	0.30
Self cure rate, extra pulmonary	0	0.04	-0.01	0.02
Rate of CD4 decline from infection to CD4=350	1	0.04	-0.01	0.23
Excess Mortality rate from HIV	2	-0.04	-0.01	-0.24
Relative risk of reinfection	2	0.03	0.01	0.49
Excess Mortality rate from HIV	3	-0.02	0.01	-0.08
TB Treatment Rate	0	-0.02	-0.02	0.03
1st-line TB treatment Cost	-	-0.01	0.01	0.00
Rate of development of active TB from presymptomatic	2	0.01	-0.00	-0.02
Self cure rate, extra pulmonary	2	0.01	-0.01	-0.06
Rate of stabilization after treatment	0	-0.01	-0.01	-0.02
Proportion TB that is extra pulmonary	2	-0.01	0.02	-0.17
Self cure rate, smear-negative pulmonary	2	0.01	-0.01	-0.07
Proportion of pulmonary TB that is smear-positive	2	0.01	-0.02	0.15
Excess Mortality rate from HIV	1	0.01	-0.05	-0.03
2nd-line treatment cost	-	-0.01	-0.01	0.01
Mortality rate from smear-positive	2	-0.00	0.02	0.28
Rate of progression from ART eligibility to on ART	2	-0.00	0.03	-0.17
Self cure rate, smear-positive	2	0.00	-0.02	-0.04
Relapse rate from active TB	0	-0.00	0.06	-0.04
ART Multiplier	-	-0.00	0.02	0.00

References

[1] Lodi S, Phillips A, Touloumi G, Geskus R, Meyer L, Thiebaut R, et al. Time From Human Immunodeficiency Virus Seroconversion to Reaching CD4+ Cell Count Thresholds . Clinical Infectious Diseases. 2011 Sep;53(8):817–825.

[2] Behr MA, Warren SA, Salamon H, Hopewell PC, Ponce de Leon A, Daley CL, et al. Transmission of Mycobacterium tuberculosis from patients smear-negative for acid-fast bacilli. The Lancet. 1999 Feb;353(9151):444–449.

[3] Vynnycky E, Fine PE. The natural history of tuberculosis: the implications of age-dependent risks of disease and the role of reinfection. Epidemiology and Infection. 1997 Oct;119(2):183–201.

[4] World Health Organization. Global Tuberculosis Report 2012; 2012.

[5] Lambert ML, Hasker E, Van Deun A, Roberfroid D, Boelaert M, Van der Stuyft P. Recurrence in tuberculosis: relapse or reinfection? The Lancet Infectious Diseases. 2003 May;3(5):282–287.

[6] Crampin AC, Mwaungulu JN, Mwaungulu FD, Mwafulirwa DT, Munthali K, Floyd S, et al. Recurrent TB: relapse or reinfection? The effect of HIV in a general population cohort in Malawi. AIDS (London, England). 2010 Jan;24(3):417–426.

[7] Horsburgh CR, O'Donnell M, Chamblee S, Moreland JL, Johnson J, Marsh BJ, et al. Revisiting rates of reactivation tuberculosis: a population-based approach. American journal of respiratory and critical care medicine. 2010 Aug;182(3):420–425.

[8] Selwyn PA, Hartel D, Lewis VA, Schoenbaum EE, Vermund SH, Klein RS, et al. A prospective study of the risk of tuberculosis among intravenous drug users with human immunodeficiency virus infection. The New England Journal of Medicine. 1989 Mar;320(9):545–550.

[9] Tiemersma EW, van der Werf MJ, Borgdorff MW, Williams BG, Nagelkerke NJD. Natural History of Tuberculosis: Duration and Fatality of Untreated Pulmonary Tuberculosis in HIV Negative Patients: A Systematic Review. PloS one. 2011;6(4):e17601.

[10] Menzies NA, Cohen T, Lin HH, Murray M, Salomon JA. Population Health Impact and Cost-Effectiveness of Tuberculosis Diagnosis with Xpert MTB/RIF: A Dynamic Simulation and Economic Evaluation. PLoS Medicine. 2012 Nov;9(11):e1001347.

[11] UNAIDS. UNAIDS Global Report 2012; 2012.

[12] Ministry of Health of the People's Republic of China. 2012 China AIDS Response Progress Report; 2012.

[13] Ayles H, Muyoyeta M, Du Toit E, Schaap A, Floyd S, Simwinga M, et al. Effect of household and community interventions on the burden of tuberculosis in southern Africa: the ZAMSTAR community-randomised trial. The Lancet. 2013 Oct;382(9899):1183–1194.

[14] Salomon JA, Vos T, Hogan DR, Gagnon M, Naghavi M, Mokdad A, et al. Common values in assessing health outcomes from disease and injury: disability weights measurement study for the Global Burden of Disease Study 2010. The Lancet. 2013 Dec;380(9859):2129–2143.

[15] Sachs JD, World Health Organization. Macroeconomics and Health. Investing in Health for Economic Development : Report. World Health Organisation (WHO); 2001.
